# The T-Cell Oncogene Tal2 Is a Target of PU.1 and Upregulated during Osteoclastogenesis

**DOI:** 10.1371/journal.pone.0076637

**Published:** 2013-09-26

**Authors:** Nadine Courtial, Christian Mücke, Stefanie Herkt, Stephan Kolodziej, Helge Hussong, Jörn Lausen

**Affiliations:** Georg-Speyer-Haus, Institute for Biomedical Research, Frankfurt, Germany; Bellvitge Biomedical Research Institute (IDIBELL), Spain

## Abstract

Transcription factors play a crucial role in regulating differentiation processes during human life and are important in disease. The basic helix-loop-helix transcription factors Tal1 and Lyl1 play a major role in the regulation of gene expression in the hematopoietic system and are involved in human leukemia. Tal2, which belongs to the same family of transcription factors as Tal1 and Lyl1, is also involved in human leukaemia. However, little is known regarding the expression and regulation of Tal2 in hematopoietic cells. Here we show that Tal2 is expressed in hematopoietic cells of the myeloid lineage. Interestingly, we found that usage of the Tal2 promoter is different in human and mouse cells. Two promoters, hP1 and hP2 drive Tal2 expression in human erythroleukemia K562 cells, however in mouse RAW cells only the mP1 promoter is used. Furthermore, we found that Tal2 expression is upregulated during oesteoclastogenesis. We show that Tal2 is a direct target gene of the myeloid transcription factor PU.1, which is a key transcription factor for osteoclast gene expression. Strikingly, PU.1 binding to the P1 promoter is conserved between mouse and human, but PU.1 binding to P2 was only detected in human K562 cells. Additionally, we provide evidence that Tal2 influences the expression of the osteoclastic differentiation gene TRACP. These findings provide novel insight into the expression control of Tal2 in hematopoietic cells and reveal a function of Tal2 as a regulator of gene expression during osteoclast differentiation.

## Introduction

The closely related basic helix-loop-helix transcription factors Tal1 (SCL1), Tal2 and Lyl1 are important regulators of normal development and differentiation. Tal1 is essential for primitive hematopoiesis and plays a role as a regulator of erythrocytic/megakaryocytic gene expression in the adult [1]. Unlike Tal1, the closely related Lyl1 protein is not needed for early hematopoietic development [2,3] and may have overlapping and independent functions with Tal1 in gene regulation [4].

The Tal2 protein consists of an N-terminal bHLH-domain, which is highly similar to Tal1 and Lyl1 and has a short C-terminus. However, the Tal2 protein is smaller than Tal1 and Lyl1, because it lacks N-terminal transactivation or repression domains. Despite these differences, Tal1, Lyl1 and Tal2 are associated with T-cell acute lymphoblastic leukaemia (T-ALL) [5]. Here, chromosomal translocations lead to the misregulation of the transcription factors, which might be causally connected to the disease [6,7,8,9,10]. *Tal2* was found at the t(7;9) (q35;q34) chromosomal translocations associated with T-ALL [6]. In contrast to its closely related family members *Tal1* and *Lyl1*, *Tal2* expression was not described in the hematopoietic system during normal hematopoietic differentiation. However, in the testis, the brain of mice and the lateral floor plate of zebrafish *Tal2* is expressed [11,12,13]. Targeted disruption of *Tal2* in the mouse leads to developmental defects in the central nervous system and to early death after birth [11]. In this study no overt defects in hematopoiesis were found in *Tal2* -/- mice. Recently, it was reported that *Tal2* is over expressed in non-small cell lung cancer tissue compared to normal lung tissue [14]. Furthermore, *Tal2* expression was connected to human epithelial ovarian cancer [15]. This raises the question if *Tal2* is causally connected to other cancer types than T-cell leukaemia.

Although *Tal2* has an important function in development and leukemia, little is known regarding the regulation of *Tal2* and its expression in different cell types. Recently, we showed that the related transcription factor *Tal1* plays a role in osteoclast differentiation [16]. This observation prompted us to examine the expression of *Tal2* during osteoclast differentiation. Osteoclasts are multinucleated bone resorbing cells, which differentiate from a myeloid progenitor upon stimulation with the cytokines macrophage-colony-stimulating factor (M-CSF) and receptor activator of NF-kB ligand (RANKL) [17,18,19,20,21,22,23]. Downstream of these cytokines osteoclast gene expression and differentiation is regulated by transcription factors such as Tal1, PU.1, MITF, AP1, MafB, CEBPbeta and NFATC [16,24,25,26,27,28]. Deregulation of osteoclast function plays a major role in human disease like osteoporosis, multiple myeloma and metastatic breast cancer [17,18,29,30].

In this study we found that *Tal2* expression is upregulated during M-CSF/RANKL induced osteoclastogenesis. Furthermore we detected Tal2 expression in the human erythroleukaemia cell line K562 and found that Tal2 is regulated from two alternative promoters in human cells. Although Tal2 expression regulation might be different between mouse and human, binding of PU.1 to Tal2 regulatory elements is conserved.

## Materials and Methods

### Bioinformatics

Analysis of the *Tal2* 5’-region for transcription factor binding sites was performed using TESS [31]. Evolutionary conserved regions between different species were defined with the help of the ECR-browser [32] using standard settings. Expression database analysis of mouse *Tal2* was performed with BioGPS [33]. Repeat elements in the 5’ region of human Tal2 were determined using the repeatmasker software (Smit, AFA, Hubley, R & Green, P. *RepeatMasker Open-3.0*. 1996-2010 http://www.repeatmasker.org).

### Cell culture and mice

RAW264.7, HEK293 and HeLa cells (ATCC) were cultured in Dulbecco’s modified eagle medium (DMEM; Gibco), K562 and U937 (ATCC) cells were cultured in RPMI medium (Gibco), both media supplemented with 10% fetal calf serum (FCS; PAA), 2 mM L-glutamine and 1% penicillin/streptomycin (Gibco). To differentiate RAW264.7 towards the osteoclast lineage, cells were cultured in the presence of 30 ng/ml recombinant murine RANK-L-TEC (R&D Systems). For differentiation of U937 cells were first treated with 0.1 µg/ml TPA to induce monocytic differentiation, after 2 days cells were treated with 50 ng/ml recombinant M-CSF (R&D Systems) and 100 ng/ml recombinant RANK-L-TEC for additional three/six days. To obtain bone marrow derived monocytes/macrophages (BMM) bone marrow cells were prepared from BL/6 mice as previously described [16].

### RNA isolation and real-time RT-PCR

Total RNA was isolated using the RNeasy mini kit (Qiagen). cDNA was synthesized using the Omniscript reverse transcriptase (Qiagen). Quantitative PCR was performed on a LightCycler 480 (Roche) using SYBR-Green PCR MasterMix (Eurogentec) and the following primers: human Tal2 forward 5´- AAAGCCTGCAACAAACGGGA-3´ and reverse 5´-TGGACCAGGTGAAGGAACCT -3´; murine Tal2 forward 5´-TGCATCAAACAGGAGTCGCT-3´ and reverse 5´-GGAAGGAACTCGGTAGTCAT-3´; human TRACP forward 5´- GGACTGAAGGGACTCCTGAAT-3´ and reverse 5´-GGTCCCTGAGCCTTTATTCC-3´; murine TRACP forward 5´-GACAAGAGGTTCCAGGAGACC-3´ and reverse 5´-GGGCTGGGGAAGTTCCAG-3´; murine DC-STAMP forward 5´-TGTATCGGCTCATCTCCTCCAT-3´ and reverse 5´-GACTCCTTGGGTTCCTTGCTT-3´; murine Cathepsin K forward 5´-ACAGCAGGATGTGGGTGTTCA-3´ and reverse 5´-GCCGAGAGATTTCATCCACCT-3´; murine MMP9 forward 5´-ACGACATAGACGGCATCCA-3´ and reverse 5´-GCTGTGGTTCAGTTGTGGTG-3´. Relative amounts of mRNA were calculated by the delta-delta-Ct method using murine GAPDH forward 5´-TCCACTCATGGCAAATTCAA-3´ and reverse 5´-TTTGATGTTAGTGGGGTCTCG-3´ or murine TBP forward 5´-GGCGGTTTGGCTAGGTTT-3´ and reverse 5´-GGGTTATCTTCACACACCATGA-3´ or human GAPDH forward 5´-GAGTCAACGGATTTGGTCGTATT-3´ and reverse 5´-GAATTTGCCATGGGTGGAAT-3´ as an internal control.

### Luciferase assay

Human and murine promoter sequences were amplified from genomic DNA. Around 500 bp of the human hP1, hP2 region and the murine mP1, mP2 region were cloned into the pGL4.10 luciferase reporter plasmid (Promega). The human *Tal2* promoter sequence was amplified from K562 genomic DNA. Constructs of 1600bp, 1030bp and 495bp were generated and cloned into the pGL4.10 luciferase reporter plasmid (Promega). Binding sites for PU.1 and GATA1 in the *Tal2* promoter constructs were mutated using the Site Directed Mutagenesis Kit (Stratagene). HEK293 cells were transfected with Metafecten (Biontex), RAW 264.7 and K562 cells were transfected with Metafecten Pro (Biontex), U937 cells were transfected with FuGENE (Promega) according the manufactures instruction. A CMV-promoter driven beta-galactosidase vector was co-transfected as a transfection control. Luciferase and beta-galactosidase activity was assayed 48 h after transfection. Firefly luciferase activity was normalized to beta-galactosidase to control for transfection efficiency.

### Western Blot

Western blot was performed according to standard procedures with an anti-Tal2 antibody (Santa Cruz sc-46266 and Abcam ab85432) and an anti-Lamin antibody or anti-Actin antibody (Abcam ab16048 and Santa Cruz sc-1615) as loading control. The SDS-loading buffer was supplemented with 1 M Urea for better detection of Tal2.

### Characterization of 5´-cDNA ends of Tal2

Total RNA was isolated from RAW264.7, K562 and U937 cells using RNeasy mini kit (Qiagen). The 5´-ends of the Tal2 gene were identified using the 5′-RACE System for rapid amplification of cDNA ends (Invitrogen). The following primers were used: murine Tal2-GSP1 (5′-AGTAGAGTTCTGTCATCC-3′) and human Tal2-GSP1 (5′-CTAAGGAATGTGGTGGC-3′) for cDNA synthesis, murine Tal2-GSP2 (5′-TCATCTGGCAGGCGGGTC-3′) and human Tal2-GSP2 (5′- AGGTTCCTTCACCTGGTCCA-3′) for the first PCR, murine Tal2-GSP3 (5′- TATAGGATCCGAAAGAGCCCCAGAATGT-3′) and human Tal2-GSP3 (5′-TATAGGATCCGTAGTTCTCAAGCAGAGTTC-3′) for the nested PCR. The final PCR products contained a SalI and a BamHI site for cloning into the pIRES-EGFP vector (Takara Bio) and were sequenced.

### Knockdown experiments with shRNA

For short hairpin interfering RNA experiments, shRNAs were cloned into psiRNA (Invivogen). shRNA oligos against murine Tal1 were designed using the ‘InvivoGen’s siRNAWizard’ program (http://www.sirnawizard.com/design.php). As control, a nonspecific shRNA was used. Sequences targeted by shRNAs: Control (5’-GTG ACC AGC GAA TAC CTG TT-3’) and murine Tal2 (5’-GTC GCT GCT CAG GGA AAC AT -3’). The integrity of the constructs was verified by sequencing.

### Chromatin immunoprecipitation

Chromatin immunoprecipitation (ChIP) assays were performed as described [34]. Sonicated chromatin from K562 or RAW264.7 cells was incubated with anti-RNAPolII (Abcam, ab5408), anti-GATA1 (Santa Cruz, sc-1233) or anti-PU.1 (SantaCruz, sc-352). Normal IgG (Santa Cruz) was used as control. DNA was amplified using the following primers: human Tal2 promoter hP1-forward 5´-CGGATACGGACTTTCCCAGG-3´ and hP1-reverse 5´-CTGGAAAGGGAGCAAGGGAG-3´; human Tal2 promoter hP2-forward 5´-GCGTCACTGATGCCTCGAAAGGT-3´ and hP2-reverse 5´-AGATGGAAAGAGAAAGGGCCCTCAG-3´; murine Tal2 promoter mP1-forward 5´-AAGGGGATCTGGAGGTGGCCA-3´ and mP1-reverse 5´-CCAAGTGCAGCAGCCAAGGAG-3´; murine Tal2 promoter mP2-forward 5´-GCTAACATTGCAGGTTCTTGGCAG-3´ and mP2-reverse 5´-TCCAGGTTCCTGCGATGAGAGAGA-3´.

## Results

### Expression of Tal2

**Figure 1:**
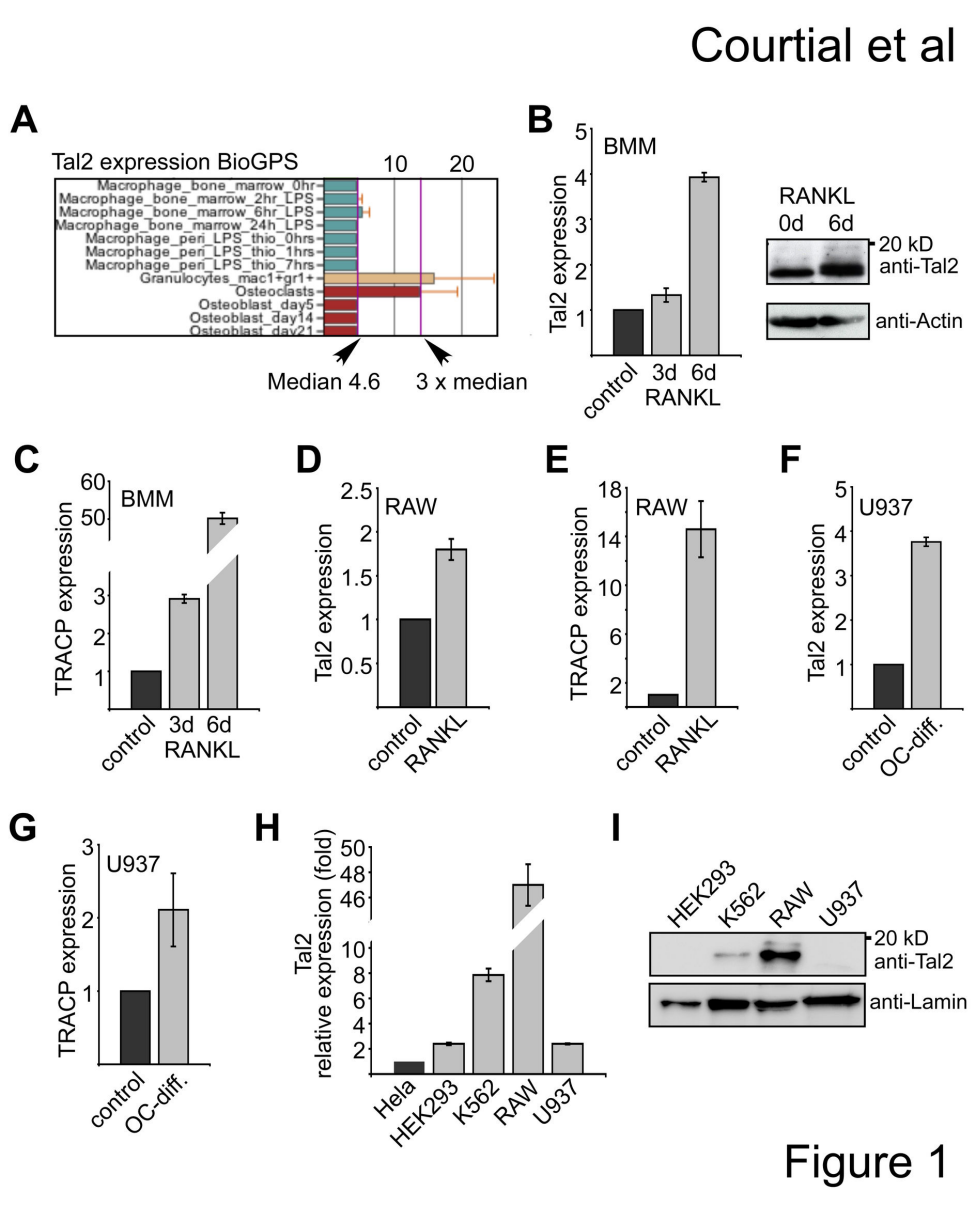
Expression of Tal2. (**A**) Expression of murine *Tal2* is higher in osteoclasts than the median expression in other cells, for example macrophages. Screenshot was taken from BioGPS (GeneAtlas MOE430, gcrma). (**B**) Expression of murine *Tal2* measured by qPCR from bone marrow derived macrophage/monocytes (BMM) cells and BMM cells differentiated towards osteoclasts for three or six days days with RANKL. Increased Tal2 protein amount was detected at day six upon treatment with RANKL by western blot (ab85432). (**C**) Expression of murine *TRACP* measured by qRT-PCR in RAW cells differentiated towards osteoclasts for three and six days with RANKL. (**D**) Expression of murine *Tal2* and measured by qRT-PCR in RAW cells upon RANKL treatment for three days. (**E**) Expression of *TRACP* in RAW cells upon RANKL treatment for three days. (**F**-**G**) Expression of *Tal2* and *TRACP* in wild type U937 cells and U937 cells differentiated towards osteoclasts by treatment with TPA for two days and three days with a combination of M-CSF and RANKL. (**H**) Comparison of the mRNA expression of *Tal2* in different cell lines by qPCR. Expression values are shown as fold compared to Hela cells. Standard deviations give the error from at least four determinations. (**I**) Western blot analysis of Tal2 protein expression in different cell lines (sc-46266).

To examine *Tal2* expression we pursued a database search using BioGPS [33]. In this publicly available data set a three times higher expression of *Tal2* was detected in osteoclasts and granulocytic cells than in macrophages of the mouse (Figure 1A). Subsequently, we analyzed *Tal2* expression in bone marrow derived monocyte macrophage cell (BMM) and upon *in vitro* osteoclast differentiation of the BMM cells. We detected a slight upregulation of *Tal2* at day three of differentiation and a 4-fold increase at day six of differentiation at the mRNA level (Figure 1B), which is in agreement with the BioGPS data. We also detected increase of Tal2 protein at day six upon differentiation (Figure 1B). Concomitantly, expression of the osteoclast differentiation gene *TRACP* was increased (Figure 1C). Similarly, we found *Tal2* upregulation during osteoclastic differentiation of the mouse monocyte macrophage RAW cell line (Figure 1D,E), when we exposed the cells to osteoclast differentiation conditions. The upregulation of *Tal2* and *TRACP* was also seen in the human U937 cell line under osteoclast differentiation conditions (Figure 1F,G). Additionally, we analyzed several cell lines of hematopoietic and non-hematopoietic origin for *Tal2* mRNA expression by quantitative PCR. *Tal2* mRNA was almost not detectable in Hela cells. Expression of *Tal2* in HEK293 cells was 2-fold higher than in Hela cells, in K562 cells *Tal2* expression was more than 7-fold higher. The highest mRNA expression was detected in RAW 264.7 cells (Figure 1H). The mRNA expression levels correlated with the Tal2 protein amounts determined by western blot with some Tal2 expression in K562 cells and high expression in RAW cells (Figure 1I). Taken together, our data show that *Tal2* is expressed in the human erythroleukaemia cell line K562 and in the mouse monocyte/macrophage cell line RAW. *Tal2* mRNA is upregulated upon RANKL induced osteoclast differentiation of mouse BMM cells and human U937 cells suggesting a role of Tal2 in this process.

### Comparative genomics of the Tal2 locus

**Figure 2:**
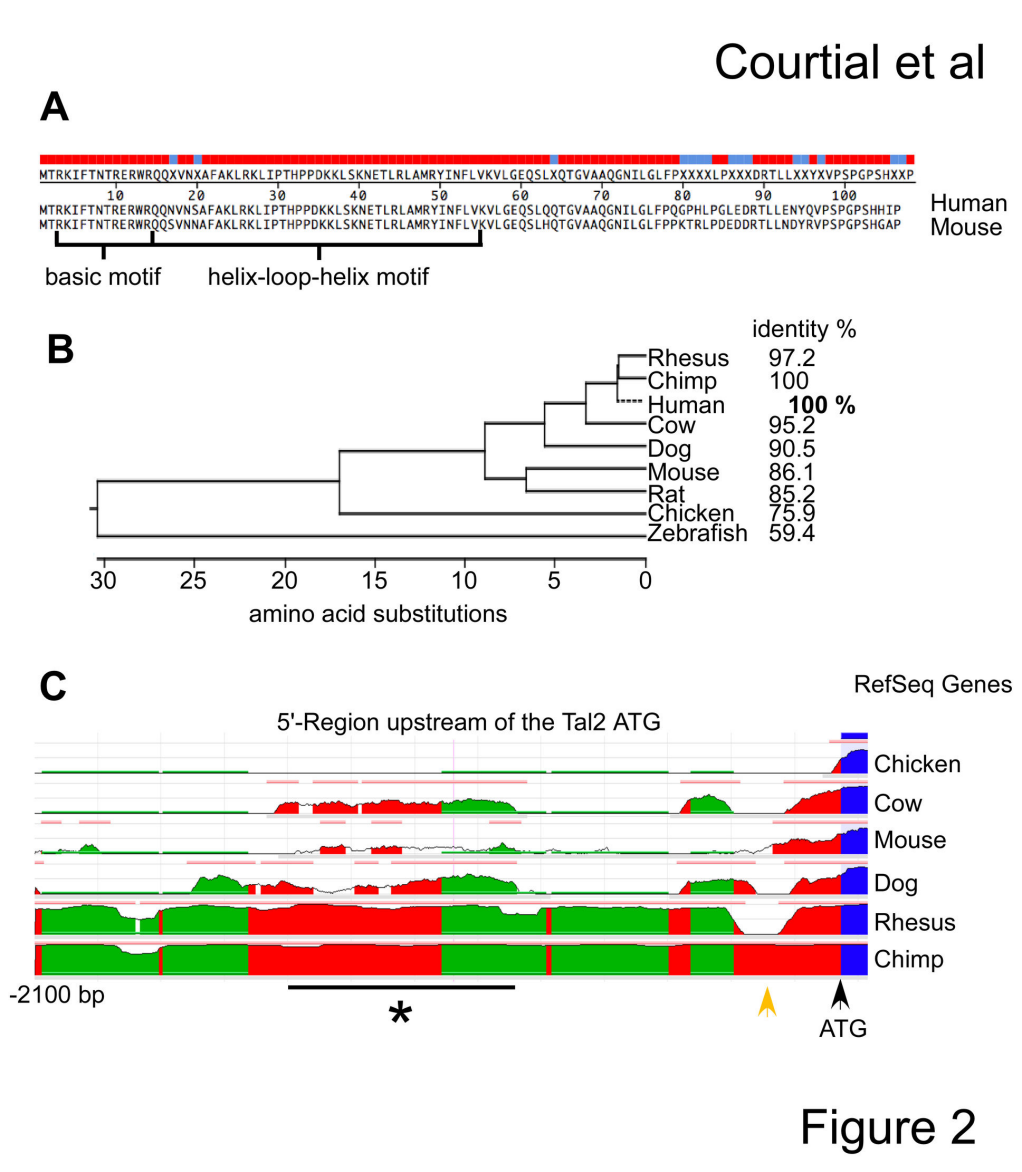
Comparison of the Tal2 locus between species. (**A**) Alignment of the human and murine Tal2 protein sequence. The colour code gives identical and divergent amino-acids, with identical amino-acids marked in red. The position of the basic motif and the helix-loop-helix motif is shown. (**B**) Comparison of the Tal2 protein sequence between different species is shown as phylogenic tree comparing the number of amino acid substitutions and as percent identity. The human sequence represents the 100% value, highlighted in bold. (**C**) Comparison of the genomic 5’-regions of *Tal2* using the ECR-browser, screenshot. The human sequence served as the reference genome. The degree of conservation is visualized by the height of the curve belonging to the corresponding species. Evolutionary conserved regions (ECRs) are shown as a bar in peach colour above the curve. The position of the ATG start codon is given (black arrow) and a region of homology loss between human and rhesus macaque is marked (orange arrow). A conserved region, except in mouse, is marked with a star.

The coding region of *Tal2* exhibits a high degree of cross species conservation. Between human and mouse most amino-acid differences are located in the C-terminus of the protein, whereas the N-terminus with the basic motif and the helix-loop-helix motif are similar (Figure 2A, Megaline-DNASTAR). Comparison of the Tal2 protein sequence relationship between different species roughly reflects their phylogenic relationship (Figure 2B). Taken together the degree of sequence conservation suggested a functional conservation of Tal2 between the species including mouse and human.

However, we found that the 5’-region in front of the ATG of *Tal2* is divergent between species. We analyzed the 5’-region of *Tal2* using the evolutionary conserved region browser (ECRbrowser) [32]. Apart from the coding region there are no evolutionary conserved regions (ECRs) detected between human and chicken (Figure 2C, upper part). The area adjacent to the ATG is conserved between cow, mouse, dog, rhesus macaque, chimp and human. Whereas the 5’ region of human and chimp is almost identical, there is a dip in conservation from human towards rhesus macaque (Figure 2C, marked with an orange arrow). Further upstream a conserved area is detected between human, chimp, rhesus macaque and cow (Figure 2C, marked with a star). The total sequence homology of the 5’-region of the *Tal2* gene is low between human and mouse (Figure 2C).

Subsequently, we scanned the 5’-region of the human Tal2 gene using the repeatmasker software (Smit, AFA, Hubley, R & Green, P. *RepeatMasker Open-3*.*0*.1996-2010 http://www.repeatmasker.org). We found three repeat elements close to the proximal promoter region of hTal2, a Line-element followed by an AluSX1#Sine and another Line-element (data not shown). These elements are also found in chimp and rhesus macaque, but not in mouse, suggesting that the introduction of transposable elements is a contributing factor to the diversity of the 5’ region between human and mouse. This result also raises the question if promoter usage and expression pattern of Tal2 is identical between the two species. Interestingly, differences in the 5’-region between human and mouse are also reflected by the presence of recorded exons in the UCSC-genome browser/database [35,36]. Whereas in human, chimp and rhesus macaque only one exon has been reported, in mouse exists another exon, more than 6500 bp 5’ of the ATG.

**Figure 3:**
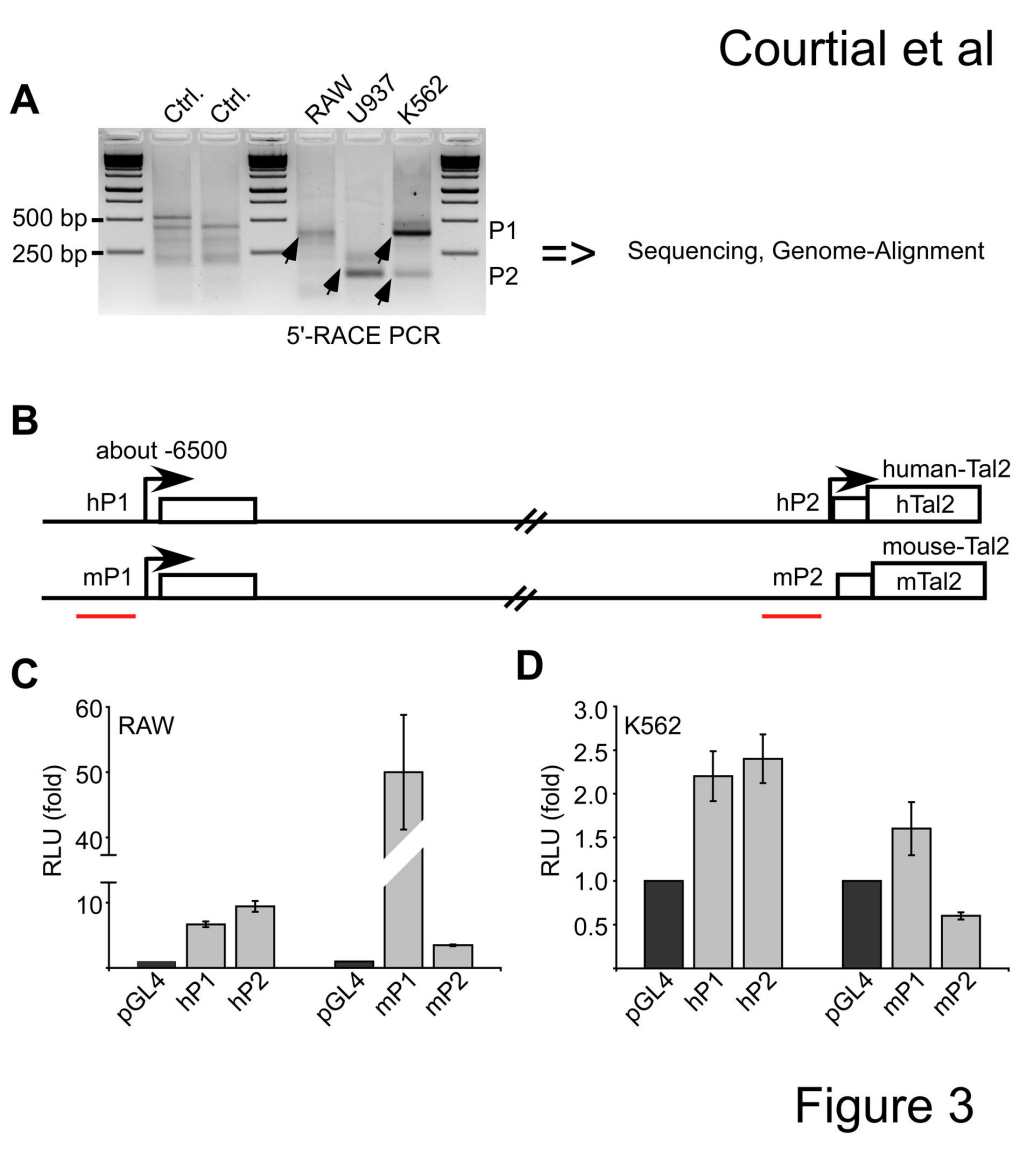
Evaluation of the Tal2 transcriptional start site in human and mouse. (**A**) 5’-RACE (rapid amplification of cDNA ends) defines transcription start sites of Tal2 in distinct cells. 5’-RACE was performed using mRNA from murine RAW, U937 and K562 cells of human origin. Upon PCR the products were analysed on a gel, sequenced and aligned to the genome. Two different start sites were defined and named the P1-promoter and the P2-promoter. Specific products are marked with an arrow. (**B**) Schematic representation of the human and mouse genomic region of Tal2. The human Tal2 gene has two alternative promoters hP1 and hP2. hP1 is located at about -6500 bp from the ATG and includes an alternative non-coding exon. In mouse RAW cells the mP1 promoter is active. The position of primer pairs for ChIP is marked. (**C**) In a luciferase experiment the promoter activity of the first 500 bp of the human hP1 and hP2 region was compared with the mouse mP1 and mP2 region in murine RAW cells. (**D**) Luciferase experiment comparing the promoter activity of the first 500 bp of the human hP1 and hP2 region with the mouse mP1 and mP2 region in human K562 cells. Values are given as fold related to the luciferase values gathered with the empty pGL4 vector. Error bars give the standard deviation of four determinations.

To gain information on the promoter usage at the Tal2 locus we mapped the 5’-mRNA end by 5’-RACE in RAW, U937 and K562 cells. Upon PCR amplification of the 5’-regions, the PCR products were sequenced and aligned to the genomes (Figure 3A). The 5’-RACE revealed different mRNA start sites of Tal2 in distinct cell lines. In mouse RAW cells we detected one transcription start site, which is in front of a non-coding exon approximately 6500 bp upstream of the ATG. This sequence was named P1 (mP1 in Figure 3B). In U937 cells of human origin the transcription starts proximal to the ATG, with no alternative exon (named hP2). Interestingly, in K562 cells we find two bands, which correspond to two transcriptional start sites. The higher band matches to an alternative promoter with an additional exon similar to the RAW cells and the lower band to a start site close to the ATG similar to U937 cells (Figure 3A). We conclude that in K562 cells Tal2 can be transcribed from two alternative promoters, which we termed as hP1 and hP2. Here, the hP1 promoter is similar to the start site used in the mouse RAW cells (Figure 3B). To further examine the differences between the human and mouse Tal2 promoters, we compared the promoter activities of the human hP1 and hP2 with the mouse mP1 and mP2 in RAW and K562 cells. For this we performed luciferase reporter assays with the first 500 bp of the hP1, hP2 and the mP1 and mP2 regions, respectively. These regions were cloned in front of the luciferase gene and the promoter activity measured in RAW and K562 cells. In RAW cells hP1 and hP2 displayed some promoter activity, however the mouse mP1 promoter displayed the highest activity, mP2 was almost inactive (Figure 3C). In K562 hP1 and hP2 showed promoter activity, where as the mouse mP1 was weakly active and mP2 showed no promoter activity (Figure 3D).

Taken together our data show differences in the regulation of Tal2 between human and mouse most notably in humans an active promoter is close to the ATG (hP2), which is not conserved and not active in mouse.

### Analysis of the human P2 Tal2 promoter

**Figure 4:**
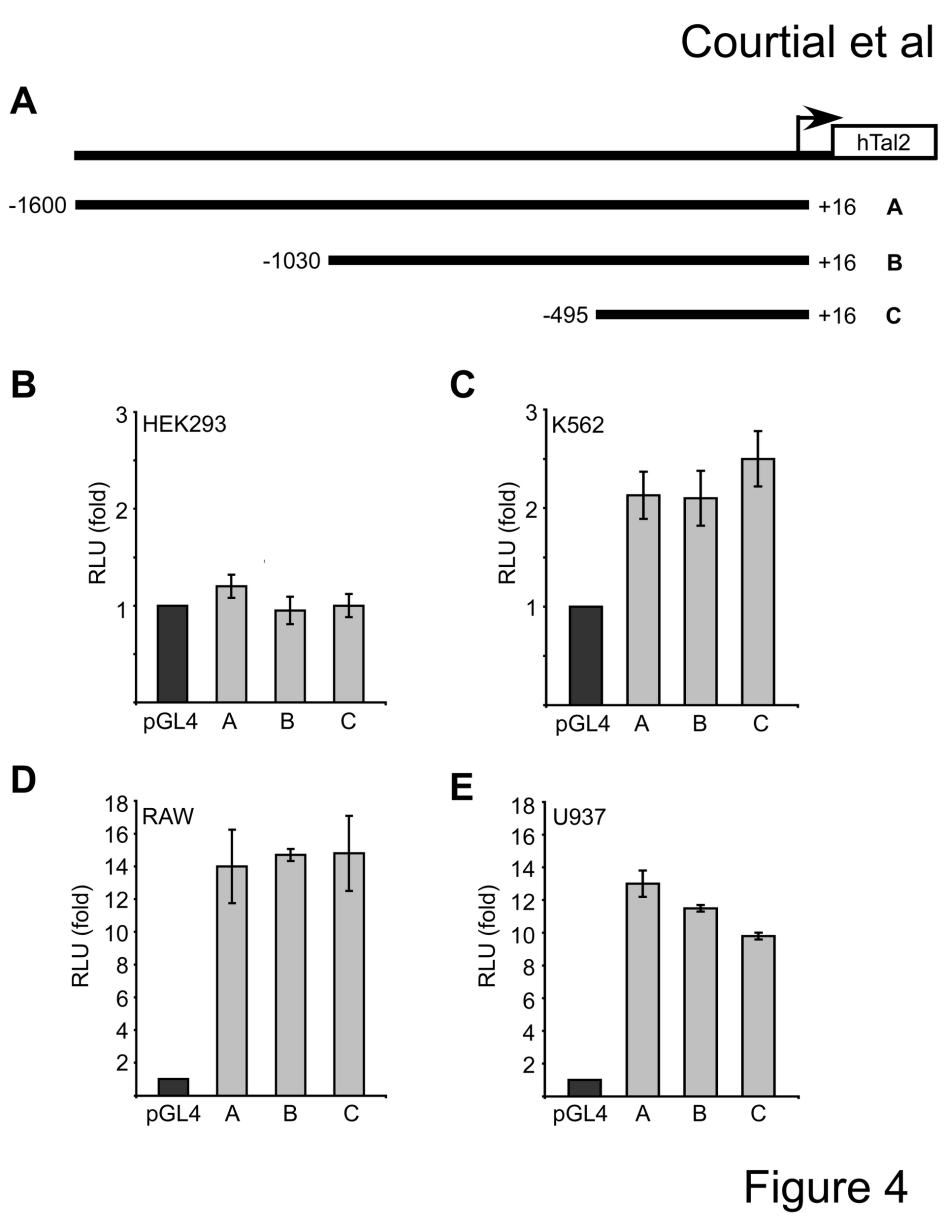
Analysis of the human Tal2 promoter activity in different cell lines. (**A**) Different length promoter luciferase constructs were established. The position of the cloned fragments with respect to to the ATG is given. The fragments were numbered from A to C. (**B**-**E**) Activity of the different length promoter constructs in HEK293, K562, RAW and U937 cells. Values were calculated as relative luciferase light units corrected for transfection efficiency by beta-galactosidase activity and are shown as fold over the activity of the empty pGL4 vector. Standard deviations were calculated from at least four determinations.

The P2 promoter of Tal2 is not conserved between human and mouse and only the human P2 promoter shows activity in K562 cells and U937 cell. For this reason we chose to further characterise the P2 promoter of human Tal2. We cloned the 5’-region of the human Tal2 gene (hTal2) from genomic DNA into the pGL4-luciferase reporter gene vector. Different length constructs were established, hTal2(-1600/+16), hTal2(-1030/+16) and hTal2(-495/+16) (Figure 4A). All promoter constructs did not show significant activity in HEK293 cells but exhibited modest promoter activity in erythroleukaemia K562 cells (Figure 4B-C). In RAW and U937 cells, which have osteoclast differentiation potential, the hTal2-promoter was active 10- to 14-fold over the empty vector (Figure 4D-E) and all promoter constructs displayed similar activity. The low promoter activity in K562 cells fits well to the observation that in these cells mostly the hP1 promoter is used. The relatively high activity in U937 cells is in agreement with the use of this hP2 promoter in U937 cells (compare with Figure 3). The high activity in RAW cells could indicate that these cells have all transcription factors present for the activation of the human P2 promoter although in these cells the mouse P1 promoter is active.

### Regulation of the hTal2 hP2 promoter by GATA1 and PU.1 in K562 cells

**Figure 5:**
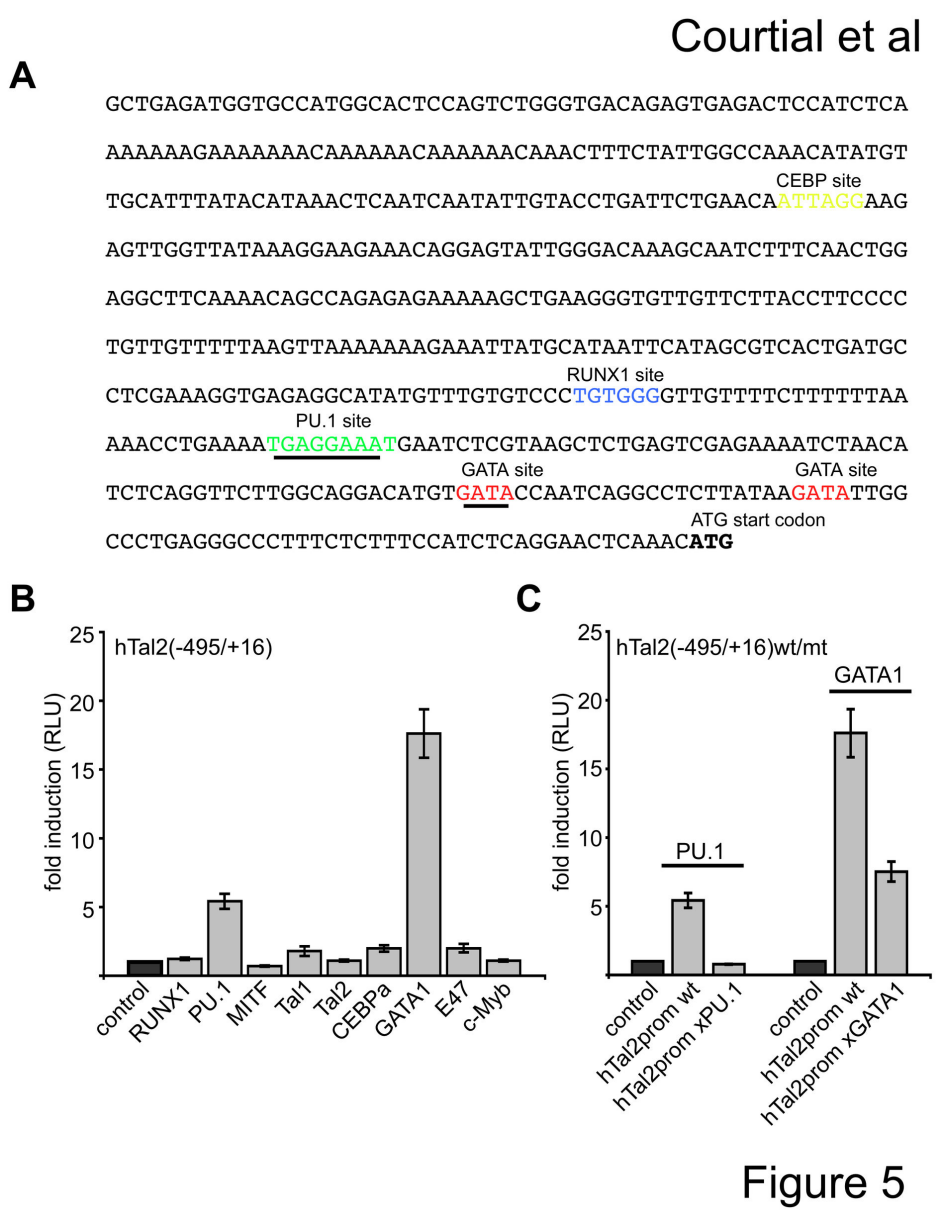
The human Tal2 promoter is activated by GATA1 and PU.1. (A) The sequence of the human *Tal2* P2-promoter is shown and identified potential transcription factor binding sites are marked in colour. (**B**) PU.1 and GATA1 activated the hTal2(-495/+16) promoter in HEK293 cells. (**C**) Mutation of the PU.1 and GATA1 site, respectively, diminish activation by the transcription factor. Wild type or mutant promoter construct were cotransfected with the given transcription factor and luciferase activity measured after 48 hours. Error bars give the standard deviation of at least six evaluations. The mutated sites are underlined.

We inspected the 5´-promoter region of hTal2 for transcription factor binding sites using TESS [31]. The hTal2(-495/+16) promoter region contains potential binding sites for the hematopoietic transcription factors PU.1, GATA, RUNX1, Tal1 and CEBP (Figure 5A). We cotransfected the hTal2(-495/+16) promoter construct with expression vectors for the different transcription factors and detected a robust induction of promoter activity by PU.1 and GATA1 (Figure 5B). Subsequently, we mutated the PU.1 site and the first GATA1 site, respectively. Mutation of the PU.1 site led to loss of activation by PU.1 and mutation of the first GATA1 site led to decreased activation by GATA1 (Figure 5C). This suggests that these sites are important for transcription factor binding.

**Figure 6:**
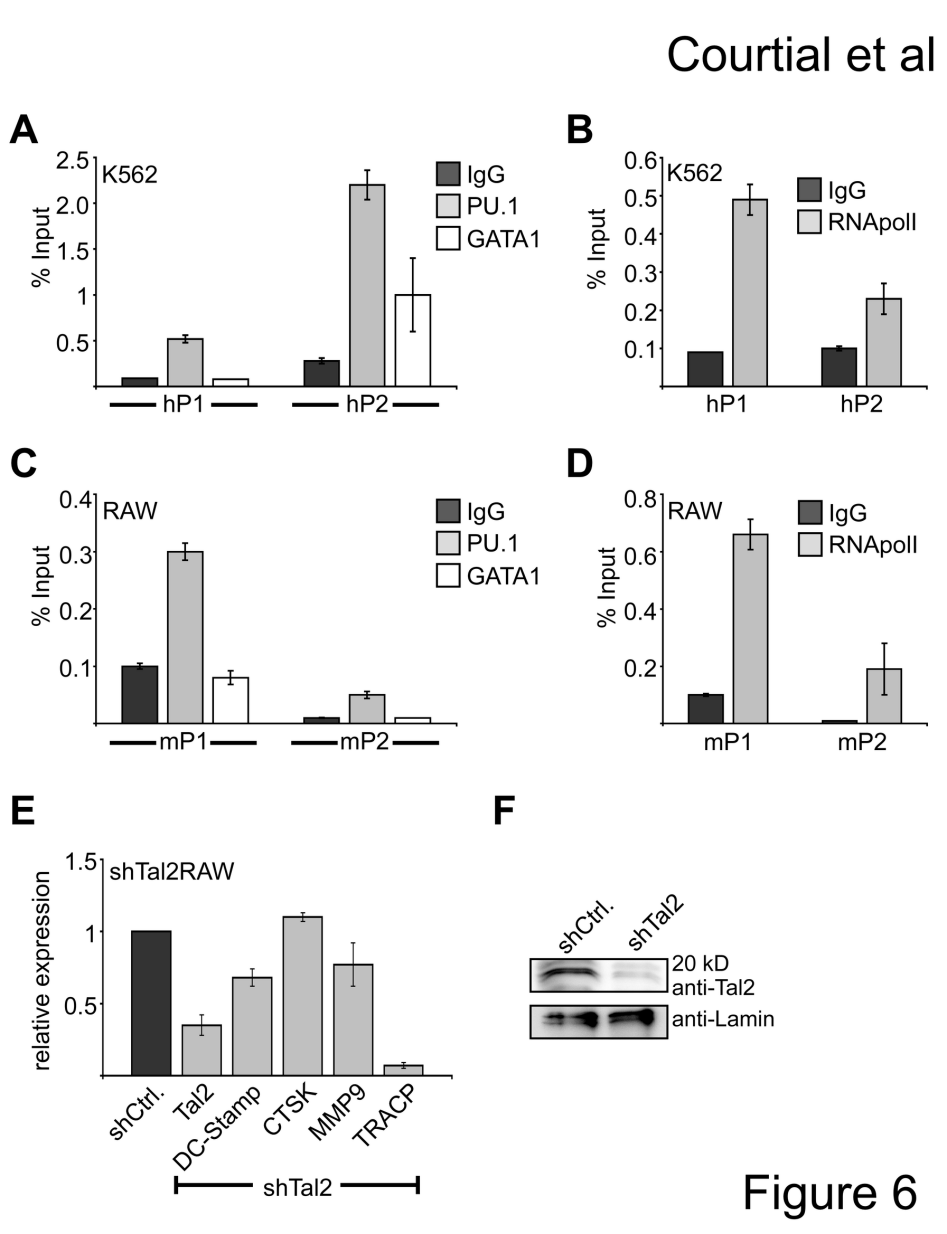
Tal2 is a direct target of PU.1 and influences expression of the osteoclast differentiation gene TRACP. (**A**) Chromatin-immunoprecipitation (ChIP) of GATA1 and PU.1 on the endogenous h*Tal2* promoter in K562 cells. PU.1 bind to some extend to the hP1 and hP2 promoter, GATA1 binding is detected at the hP2 promoter. (**B**) Chromatin immunoprecipitation in K562 cells using an antibody against RNA-polymeraseII shows binding of RNA-polymeraseII to hP1 but less to hP2 of the human *Tal2* gene. (**C**) Chromatin immunoprecipitation in RAW cells using an antibody against PU.1 shows binding of PU.1 to mP1 but to a less extend to the mP2 region of the murine *Tal2* gene. GATA1 does not bind to the mP1 or mP2 promoter. (**D**) Chromatin immunoprecipitation in RAW cells using an antibody against RNA-polymeraseII shows binding of RNA-polymeraseII to mP1 but less to mP2 of the murine *Tal2* gene. (**E**) Knockdown of *Tal2* decreases *TRACP* expression. qRT-PCR was performed from RAW cells upon knockdown of *Tal2* by shRNA. Standard deviations give the error from at least four determinations. (**F**) Western blot analysis showing the Tal2 knock down on the protein level in RAW cells.

In agreement with the luciferase data we detected binding of GATA1 and PU.1 to the endogenous hTal2 hP2 promoter by chromatin immunoprecipitation, verifying that hTal2 is a direct target gene of GATA1 and PU.1 in K562 cells (Figure 6A). PU.1 binding was detected at both hP1 and hP2 promoter regions, but mostly at the hP2 promoter, whereas GATA1 binding was exclusively found at hP2 (Figure 6A). Binding of RNA-polymeraseII was mostly detected at hP1 and to a lesser extend at hP2 (Figure 6B), which fits to the RACE data (Figure 3A). When we compared the human and mouse sequence we found that the PU.1 binding site is not present in the murine mP2 promoter, however there is a PU.1 binding site in the mP1 promoter of the murine Tal2 gene. Accordingly, in murine RAW cells we detected PU.1 binding to the mP1 promoter of the mouse Tal2 gene, but not to the mP2 promoter (Figure 6C). In RAW cells no binding of GATA1 was detected at the mP1 or mP2 promoter. Furthermore, we found RNA-polymeraseII present mostly at the P1 promoter, which is in line with the use of the P1 promoter in RAW cells (Figure 6D). Taken together, although the promoter sites differ, PU.1 targets the murine and the human Tal2 gene and GATA1 binding could only be detected in the erythroleukemia cell line K562.

### Knockdown of Tal2 influences TRACP expression

Tal2 expression was high in the monocyte/macrophage RAW cells, which can be induced to differentiate into osteoclast cells. This is in agreement to the data from the Symatlas expression databank indicating that Tal2 is expressed highest in osteoclast cells compared to other tissues (Figure 1). To gather information if Tal2 is involved in osteoclast related functions, we performed an shRNA mediated knockdown of Tal2 (Figure 6E). Tal2 knock down was detectable at the mRNA and protein level (Figure 6E,F). When we analyzed the expression of genes associated with osteoclast differentiation in the shTal2 cells we found a significant decrease of the expression of the tartrate resistant acid phosphatase gene *TRACP* (Figure 6E). This suggests that Tal2 takes part in the control of the gene expression program during osteoclastogenesis.

## Discussion

### Expression of Tal2

Ectopic Tal2 expression is associated with acute lymphatic T-cell leukaemia in humans. However *Tal2* expression was not reported in normal hematopoietic cells of human or mouse origin. In the murine model Tal2 expression was detected mostly in the brain but not in hematopoietic cells and in the Tal2 knockout mice no overt hematopoietic defect was detected. Our data show that *Tal2* mRNA and protein is expressed in a number of cell lines of myeloid origin such as the human erythroleukaemia cell line K562 and the murine monocyte/macrophage cell line RAW. Furthermore, we found *Tal2* also in mouse primary bone marrow monocyte/macrophage (BMM) cells. We detected upregulation of *Tal2* during osteoclast differentiation of RAW cells and BMM cells of murine origin and in U937 cells of human origin. This is in agreement with our finding that the human *Tal2* promoter is active in cells with osteoclast differentiation potential. This indicates that Tal2, similar to its family member Tal1, has a functional role in osteoclast differentiation [16].

### Differences between human and murine Tal2

The degree of sequence conservation on the protein level suggests a functional conservation between species, including mouse and human. Accordingly, the analysis of Tal2 function and expression in the mouse e.g. expression in the brain and no expression in the hematopoietic system, was translated to the human system in most publications regarding Tal2. We found that human Tal2 can be transcribed from two alternative promoters hP1, which is about 6500 bp upstream from the ATG and hP2, which is proximal to the ATG. Promoter usage seems to be cell type dependent. In U937 cells we only detected transcripts from the hP2 promoters, whereas in K562 cells both promoters are used, but the hP1 promoter seemed to have higher activity. In mouse RAW cells only transcripts from the mP1 promoter were detected and the mP2 promoter was inactive in contrast to the hP2 promoter.

Interestingly, in human K562 cells the region proximal of the ATG is used as a promoter and includes a PU.1 binding site, this site is not conserved and not used in the mouse. In K562 erythroleukaemia cells we detected PU.1 binding at the hP1 and hP2 site and additionally GATA1 binding was detected at the hP2 promoter. This could indicate that the hP2 promoter plays a role in cells where also PU.1 and GATA1 are involved such as erythroid/megakaryocytic differentiation.

In murine RAW cells the mP2 region has no promoter activity and neither PU.1 nor GATA1 binding could be detected. However, at the mP1 promoter PU.1 binding was detected in front of an additional non-coding exon 6500bp upstream of the ATG. In contrast to K562 cells GATA1 binding was not found at the mP1 or mP2 promoter in RAW cells. The differences between human and mouse could be caused by the introduction of transposable elements 5’ of the ATG in the human sequence as we find three of these elements in the human gene but not in the mouse. Interestingly transposons are frequently found in regulatory regions like promoters [37].

Our finding that PU.1 binds to the Tal2 promoter in mouse and human cells is in line with our observation that Tal2 is expressed in the myeloid lineage, because PU.1 is a master regulator of myeloid differentiation [38]. Moreover, PU.1 plays a crucial role in osteoclast differentiation [39,40] and gene expression [41,42,43]. In human an additional hP2 promoter is active, which harbours binding sites for hematopoietic transcription factors such as PU.1, GATA1 and RUNX1. This is a hint that expression of Tal2 might be different in human and mouse especially in cells where the hP2 promoter is activated.

### Potential molecular function of Tal2

Upon knockdown of murine Tal2 we observed a reduced expression of TRACP, which encodes for an important enzyme of osteoclast function and differentiation. Other important osteoclast specific genes like DC-STAMP and CTSK remain unchanged. For further analysis of Tal2 function in osteoclast differentiation a conditional Tal2 knockout in the osteoclast lineage would be highly desired. The finding that Tal2 can influence the expression of osteoclast genes raises the question how Tal2 acts at the molecular level on gene expression. Because Tal2 is only a small protein consisting of a conserved bHLH and a less conserved short C-terminus with no defined repressor or activator domain, we speculate that Tal2 could function by dimerising to other bHLH factors like E47 or Tal1 in osteoclast differentiation. An important hint may come from data showing that Tal2 is not able to bind DNA alone but may act by dimerisiation with other bHLH protein like E47 [44]. In this regard our recent finding that Tal1 influences osteoclast specific genes [16] would be in line with a function of Tal2 in conjunction with Tal1.

In conclusion *Tal2* expression is upregulated during osteoclast differentiation in the mouse and human system and may take part in the regulation of osteoclast genes. Despite diversity in promoter usage, PU.1 binding to mouse and human Tal2 regulatory elements is maintained. However, other aspects of Tal2 expression regulation might be different between species. Tal2 expression is linked to T-cell leukaemia, we also detected Tal2 expression in the erythroleukaemia cell line K562, and thus it might be involved in this form of leukaemia. The further analysis of *Tal2* expression and function in the human system should reveal differences to the mouse model and shed light on the potential role of *Tal2* in osteoclast related disease and leukaemia.

## References

[1] Lécuyer E, Hoang T (2004) SCL: from the origin of hematopoiesis to stem cells and leukemia. Exp Hematol 32: 11-24. doi:10.1016/j.exphem.2003.10.010. PubMed: 14725896. 14725896 10.1016/j.exphem.2003.10.010

[2] Capron C, Lécluse Y, Kaushik AL, Foudi A, Lacout C et al. (2006) The SCL relative LYL-1 is required for fetal and adult hematopoietic stem cell function and B-cell differentiation. Blood 107: 4678-4686. doi:10.1182/blood-2005-08-3145. PubMed: 16514064. 16514064 10.1182/blood-2005-08-3145

[3] Chan WY, Follows GA, Lacaud G, Pimanda JE, Landry JR et al. (2007) The paralogous hematopoietic regulators Lyl1 and Scl are coregulated by Ets and GATA factors, but Lyl1 cannot rescue the early Scl-/- phenotype. Blood 109: 1908-1916. doi:10.1182/blood-2006-05-023226. PubMed: 17053063. 17053063 10.1182/blood-2006-05-023226

[4] Curtis DJ, Salmon JM, Pimanda JE (2012) Concise Review: Blood Relatives: Formation and regulation of hematopoietic stem cells by the basic helix-loop-helix transcription factors stem cell leukemia and lymphoblastic leukemia-derived sequence 1. Stem Cells 30: 1053-1058. doi:10.1002/stem.1093. PubMed: 22593015. 22593015 10.1002/stem.1093

[5] Baer R (1993) TAL1, TAL2 and LYL1: a family of basic helix-loop-helix proteins implicated in T cell acute leukaemia. Semin Cancer Biol 4: 341-347. PubMed: 8142619. 8142619

[6] Xia Y, Brown L, Yang CY, Tsan JT, Siciliano MJ et al. (1991) TAL2, a helix-loop-helix gene activated by the (7;9)(q34;q32) translocation in human T-cell leukemia. Proc Natl Acad Sci U S A 88: 11416-11420. doi:10.1073/pnas.88.24.11416. PubMed: 1763056. 1763056 10.1073/pnas.88.24.11416 PMC53146

[7] Begley CG, Aplan PD, Davey MP, Nakahara K, Tchorz K et al. (1989) Chromosomal translocation in a human leukemic stem-cell line disrupts the T-cell antigen receptor delta-chain diversity region and results in a previously unreported fusion transcript. Proc Natl Acad Sci U S A 86: 2031-2035. doi:10.1073/pnas.86.6.2031. PubMed: 2467296. 2467296 10.1073/pnas.86.6.2031 PMC286840

[8] Chen Q, Cheng JT, Tasi LH, Schneider N, Buchanan G et al. (1990) The tal gene undergoes chromosome translocation in T cell leukemia and potentially encodes a helix-loop-helix protein. EMBO J 9: 415-424. PubMed: 2303035. 2303035 10.1002/j.1460-2075.1990.tb08126.x PMC551682

[9] Finger LR, Kagan J, Christopher G, Kurtzberg J, Hershfield MS et al. (1989) Involvement of the TCL5 gene on human chromosome 1 in T-cell leukemia and melanoma. Proc Natl Acad Sci U S A 86: 5039-5043. doi:10.1073/pnas.86.13.5039. PubMed: 2740341. 2740341 10.1073/pnas.86.13.5039 PMC297552

[10] Mellentin JD, Smith SD, Cleary ML (1989) lyl-1, a novel gene altered by chromosomal translocation in T cell leukemia, codes for a protein with a helix-loop-helix DNA binding motif. Cell 58: 77-83. doi:10.1016/0092-8674(89)90404-2. PubMed: 2752424. 2752424 10.1016/0092-8674(89)90404-2

[11] Bucher K, Sofroniew MV, Pannell R, Impey H, Smith AJ et al. (2000) The T cell oncogene Tal2 is necessary for normal development of the mouse brain. Dev Biol 227: 533-544. doi:10.1006/dbio.2000.9920. PubMed: 11071772. 11071772 10.1006/dbio.2000.9920

[12] Mori S, Sugawara S, Kikuchi T, Tanji M, Narumi O et al. (1999) The leukemic oncogene tal-2 is expressed in the developing mouse brain. Brain research. Mol Brain Res 64: 199-210. doi:10.1016/S0169-328X(98)00323-4. PubMed: 9931488. 9931488 10.1016/s0169-328x(98)00323-4

[13] Pinheiro P, Gering M, Patient R (2004) The basic helix-loop-helix transcription factor, Tal2, marks the lateral floor plate of the spinal cord in zebrafish. Gene Expr Patterns GEP 4: 85-92 doi:10.1016/S1567-133X(03)00145-5. PubMed: 14678833.

[14] Guo NL, Wan YW, Tosun K, Lin H, Msiska Z et al. (2008) Confirmation of gene expression-based prediction of survival in non-small cell lung cancer. Clin Cancer Res Off J Am Assoc Cancer Res 14: 8213-8220. doi:10.1158/1078-0432.CCR-08-0095. PubMed: 19088038. 10.1158/1078-0432.CCR-08-0095 PMC2605664 19088038

[15] Guo Y, Fu P, Zhu H, Reed E, Remick SC et al. (2012) Correlations among ERCC1, XPB, UBE2I, EGF, TAL2 and ILF3 revealed by gene signatures of histological subtypes of patients with epithelial ovarian cancer. Oncol Rep 27: 286-292. PubMed: 21971700. 21971700 10.3892/or.2011.1483

[16] Courtial N, Smink JJ, Kuvardina ON, Leutz A, Göthert JR et al. (2012) Tal1 regulates osteoclast differentiation through suppression of the master regulator of cell fusion DC-STAMP. FASEB J 26: 523-532. doi:10.1096/fj.11-190850. PubMed: 21990371. 21990371 10.1096/fj.11-190850

[17] Boyle WJ, Simonet WS, Lacey DL (2003) Osteoclast differentiation and activation. Nature 423: 337-342. doi:10.1038/nature01658. PubMed: 12748652. 12748652 10.1038/nature01658

[18] Novack DV, Teitelbaum SL (2008) The osteoclast: friend or foe? Annu Rev Pathol 3: 457-484. doi:10.1146/annurev.pathmechdis.3.121806.151431. PubMed: 18039135. 18039135 10.1146/annurev.pathmechdis.3.121806.151431

[19] Yavropoulou MP, Yovos JG (2008) Osteoclastogenesis--current knowledge and future perspectives. J Musculoskelet Neuronal Interact 8: 204-216. PubMed: 18799853. 18799853

[20] Teitelbaum SL, Ross FP (2003) Genetic regulation of osteoclast development and function. Nat Rev Genet 4: 638-649. doi:10.1038/nrg1122. PubMed: 12897775. 12897775 10.1038/nrg1122

[21] Asagiri M, Takayanagi H (2007) The molecular understanding of osteoclast differentiation. Bone 40: 251-264. doi:10.1016/j.bone.2006.09.023. PubMed: 17098490. 17098490 10.1016/j.bone.2006.09.023

[22] Dougall WC, Glaccum M, Charrier K, Rohrbach K, Brasel K et al. (1999) RANK is essential for osteoclast and lymph node development. Genes Dev 13: 2412-2424. doi:10.1101/gad.13.18.2412. PubMed: 10500098. 10500098 10.1101/gad.13.18.2412 PMC317030

[23] Kong YY, Yoshida H, Sarosi I, Tan HL, Timms E et al. (1999) OPGL is a key regulator of osteoclastogenesis, lymphocyte development and lymph-node organogenesis. Nature 397: 315-323. doi:10.1038/16852. PubMed: 9950424. 9950424 10.1038/16852

[24] Takayanagi H, Kim S, Koga T, Nishina H, Isshiki M et al. (2002) Induction and activation of the transcription factor NFATc1 (NFAT2) integrate RANKL signaling in terminal differentiation of osteoclasts. Dev Cell 3: 889-901. doi:10.1016/S1534-5807(02)00369-6. PubMed: 12479813. 12479813 10.1016/s1534-5807(02)00369-6

[25] Ishida N, Hayashi K, Hoshijima M, Ogawa T, Koga S et al. (2002) Large scale gene expression analysis of osteoclastogenesis in vitro and elucidation of NFAT2 as a key regulator. J Biol Chem 277: 41147-41156. doi:10.1074/jbc.M205063200. PubMed: 12171919. 12171919 10.1074/jbc.M205063200

[26] Asagiri M, Sato K, Usami T, Ochi S, Nishina H et al. (2005) Autoamplification of NFATc1 expression determines its essential role in bone homeostasis. J Exp Med 202: 1261-1269. doi:10.1084/jem.20051150. PubMed: 16275763. 16275763 10.1084/jem.20051150 PMC2213228

[27] Kim K, Kim JH, Lee J, Jin HM, Kook H et al. (2007) MafB negatively regulates RANKL-mediated osteoclast differentiation. Blood 109: 3253-3259. doi:10.1182/blood-2006-09-048249. PubMed: 17158225. 17158225 10.1182/blood-2006-09-048249

[28] Smink JJ, Bégay V, Schoenmaker T, Sterneck E, de Vries TJ et al. (2009) Transcription factor C/EBPbeta isoform ratio regulates osteoclastogenesis through MafB. EMBO J 28: 1769-1781. doi:10.1038/emboj.2009.127. PubMed: 19440205. 19440205 10.1038/emboj.2009.127 PMC2685610

[29] Helfrich MH (2003) Osteoclast diseases. Microsc Res Tech 61: 514-532. doi:10.1002/jemt.10375. PubMed: 12879419. 12879419 10.1002/jemt.10375

[30] Sambrook P, Cooper C (2006) Osteoporosis. Lancet 367: 2010-2018. doi:10.1016/S0140-6736(06)68891-0. PubMed: 16782492. 16782492 10.1016/S0140-6736(06)68891-0

[31] Schug J (2008) Using TESS to predict transcription factor binding sites in DNA sequence. Curr Protoc Bioinforma Chapter 2: Unit 2.6: Unit 2 6. PubMed: 18428685 10.1002/0471250953.bi0206s21 18428685

[32] Ovcharenko I, Nobrega MA, Loots GG, Stubbs L (2004) ECR Browser: a tool for visualizing and accessing data from comparisons of multiple vertebrate genomes. Nucleic Acids Res 32: W280-W286. doi:10.1093/nar/gkh355. PubMed: 15215395. 15215395 10.1093/nar/gkh355 PMC441493

[33] Wu C, Orozco C, Boyer J, Leglise M, Goodale J et al. (2009) BioGPS: an extensible and customizable portal for querying and organizing gene annotation resources. Genome Biol 10: R130. doi:10.1186/gb-2009-10-11-r130. PubMed: 19919682. 19919682 10.1186/gb-2009-10-11-r130 PMC3091323

[34] Herglotz J, Kuvardina ON, Kolodziej S, Kumar A, Hussong H et al. (2012) Histone arginine methylation keeps RUNX1 target genes in an intermediate state. Oncogene. 10.1038/onc.2012.274 22777353

[35] Dreszer TR, Karolchik D, Zweig AS, Hinrichs AS, Raney BJ et al. (2012) The UCSC Genome Browser database: extensions and updates 2011. Nucleic Acids Res 40: D918-D923. doi:10.1093/nar/gkr1055. PubMed: 22086951. 22086951 10.1093/nar/gkr1055 PMC3245018

[36] Fujita PA, Rhead B, Zweig AS, Hinrichs AS, Karolchik D et al. (2011) The UCSC Genome Browser database. Update 2011 Nucleic acids research 39: D876-882. 10.1093/nar/gkq963 PMC3242726 20959295

[37] Conley AB, Piriyapongsa J, Jordan IK (2008) Retroviral promoters in the human genome. Bioinformatics 24: 1563-1567. doi:10.1093/bioinformatics/btn243. PubMed: 18535086. 18535086 10.1093/bioinformatics/btn243

[38] Rosenbauer F, Tenen DG (2007) Transcription factors in myeloid development: balancing differentiation with transformation. Nat Rev Immunol 7: 105-117. doi:10.1038/nri2024. PubMed: 17259967. 17259967 10.1038/nri2024

[39] Tondravi MM, McKercher SR, Anderson K, Erdmann JM, Quiroz M et al. (1997) Osteopetrosis in mice lacking haematopoietic transcription factor PU.1. Nature 386: 81-84. doi:10.1038/386081a0. PubMed: 9052784. 9052784 10.1038/386081a0

[40] Tsuneto M, Tominaga A, Yamazaki H, Yoshino M, Orkin SH et al. (2005) Enforced expression of PU.1 rescues osteoclastogenesis from embryonic stem cells lacking Tal-1. Stem Cells 23: 134-143. doi:10.1634/stemcells.2004-0154. PubMed: 15625130. 15625130 10.1634/stemcells.2004-0154

[41] Luchin A, Suchting S, Merson T, Rosol TJ, Hume DA et al. (2001) Genetic and physical interactions between Microphthalmia transcription factor and PU.1 are necessary for osteoclast gene expression and differentiation. J Biol Chem 276: 36703-36710. doi:10.1074/jbc.M106418200. PubMed: 11481336. 11481336 10.1074/jbc.M106418200

[42] Hu R, Sharma SM, Bronisz A, Srinivasan R, Sankar U et al. (2007) Eos, MITF, and PU.1 recruit corepressors to osteoclast-specific genes in committed myeloid progenitors. Mol Cell Biol 27: 4018-4027. doi:10.1128/MCB.01839-06. PubMed: 17403896. 17403896 10.1128/MCB.01839-06 PMC1900027

[43] Sharma SM, Bronisz A, Hu R, Patel K, Mansky KC et al. (2007) MITF and PU.1 recruit p38 MAPK and NFATc1 to target genes during osteoclast differentiation. J Biol Chem 282: 15921-15929. doi:10.1074/jbc.M609723200. PubMed: 17403683. 17403683 10.1074/jbc.M609723200

[44] Xia Y, Hwang LY, Cobb MH, Baer R (1994) Products of the TAL2 oncogene in leukemic T cells: bHLH phosphoproteins with DNA-binding activity. Oncogene 9: 1437-1446. PubMed: 8152805. 8152805

